# Molecular determinants of drug-specific sensitivity for epidermal growth factor receptor (EGFR) exon 19 and 20 mutants in non-small cell lung cancer

**DOI:** 10.18632/oncotarget.3472

**Published:** 2015-02-28

**Authors:** Igor F. Tsigelny, Jennifer J. Wheler, Jerry P. Greenberg, Valentina L. Kouznetsova, David J. Stewart, Lyudmila Bazhenova, Razelle Kurzrock

**Affiliations:** ^1^ Center for Personalized Cancer Therapy, Moores UCSD Cancer Center, La Jolla, CA, USA; ^2^ San Diego Supercomputer Center, University of California, San Diego, La Jolla, CA, USA; ^3^ Department of Neurosciences, University of California, San Diego, La Jolla, CA, USA; ^4^ M.D. Anderson Cancer Center, University of Texas, Houston, TX, USA; ^5^ Department of Medicine, University of Ottawa, Ottawa, Canada

**Keywords:** EGFR, adenocarcinoma, lung cancer, reversible TKI inhibitors, irreversible TKI inhibitors

## Abstract

We hypothesized that aberrations activating epidermal growth factor receptor (EGFR) via dimerization would be more sensitive to anti-dimerization agents (e.g., cetuximab). EGFR exon 19 abnormalities (L747_A750del; deletes amino acids LREA) respond to reversible EGFR kinase inhibitors (TKIs). Exon 20 in-frame insertions and/or duplications (codons 767 to 774) and T790M mutations are clinically resistant to reversible/some irreversible TKIs. Their impact on protein function/therapeutic actionability are not fully elucidated.

In our study, the index patient with non-small cell lung cancer (NSCLC) harbored EGFR D770_P772del_insKG (exon 20). A twenty patient trial (NSCLC cohort) (cetuximab-based regimen) included two participants with EGFR TKI-resistant mutations ((i) exon 20 D770>GY; and (ii) exon 19 LREA plus exon 20 T790M mutations). Structural modeling predicted that EGFR exon 20 anomalies (D770_P772del_insKG and D770>GY), but not T790M mutations, stabilize the active dimer configuration by increasing the interaction between the kinase domains, hence sensitizing to an agent preventing dimerization. Consistent with predictions, the two patients harboring D770_P772del_insKG and D770>GY, respectively, responded to an EGFR antibody (cetuximab)-based regimen; the T790M-bearing patient showed no response to cetuximab combined with erlotinib. *In silico* modeling merits investigation of its ability to optimize therapeutic selection based on structural/functional implications of different aberrations within the same gene.

## INTRODUCTION

Patients suffering from lung cancers that harbor EGFR-sensitive mutations are responsive to reversible EGFR tyrosine kinase inhibitors (TKIs) such as erlotinib [1, 2]. In about 60% of cases initially sensitive to reversible EGFR inhibitors, the T790M mutation emerges; these are generally resistant to both reversible and some irreversible TKIs (e.g., afatinib) in the clinic [3-6]. In other patients (~4 to ~13 percent each), MET and HER2 amplification are operative in resistance [7, 8]. A small, but important, subgroup of patients carry EGFR exon 20 insertion mutations (in-frame insertions and/or duplications of 3 to 21 base pairs clustered between codons 767 and 774 in EGFR exon 20), which are known to be generally resistant to both reversible and new irreversible TKIs in the clinic [3].

The extracellular antibody cetuximab can also target EGFR. It functions by partially blocking the EGFR ligand-binding domain and sterically hindering exposure of the dimerization domain, hence decreasing docking of the two monomeric EGFRs to each other and, in this way, attenuating dimerization-dependent EGFR activity [9]. Antitumor activity of cetuximab is observed in patients with EGFR wild-type colorectal and head and neck cancers, but not in many other tumor types [10-12]. The mechanism of action of wild-type EGFR is primarily driven by receptor dimerization followed by kinase activation [13]. Shan and colleagues have postulated that EGFR L834R exon 21 mutations (also called L858R) counteract the intrinsic disorder in the EGFR kinase and, therefore, facilitate dimerization, which in turn promotes kinase activation [2].

To date, the exact mechanisms by which each of the EGFR mutations mediates pathway activation and carcinogenesis remains incompletely elucidated. Further, there are multiple reversible and irreversible EGFR TKI inhibitors and antibodies available for clinical use, but matching patients with an individual agent occurs largely based on empiric data. We therefore modeled the structure of EGFR mutants in patients with non-small cell lung cancer (NSCLC) and correlated clinical outcomes with predicted functional implications. These studies form a proof-of-principle demonstration of the ability of *in silico* modeling to be used to choose therapy for individuals, and suggest that further investigation in larger cohorts of patients is needed.

## RESULTS

### Structural modeling

### D770 region (insertion exon 20) mutants

**Figure 1:**
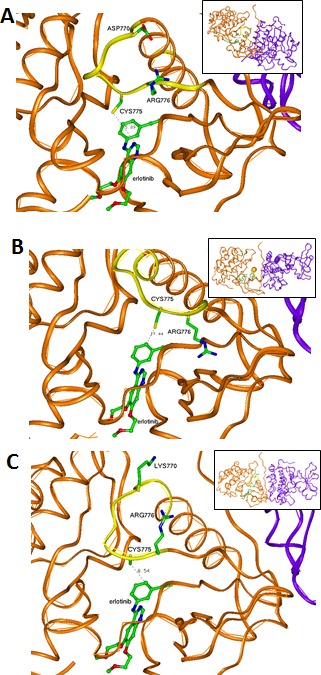
Interaction between drugs (ball-and-stick presentation) and kinase domains of EGFR (brown-the first domain and violet-the second domain) (A) LREA mutant EGFR interacting with erlotinib. CYS775 can have a hydrophobic interaction with the drug; the exon 20 structure of the exon 19 LREA EGFR mutant is similar to that found in wt EGFR. The region of the first domain that was not changed due to this mutations (ribbon) is in yellow. (B) Model of a D770>GY mutant. Absence of the negative ASP770 affects electrostatic interaction between the kinase subunits. CYS775 is moved by this mutation far from the front atoms of the erlotinib causing its unstable positioning in the ATP-binding pocket. The region of the first domain that was changed due to the mutations (ribbon) is in yellow. (C) Model of D770_P772del_insKG mutant. LYS770 introduced by the mutation and absence of the ASP770 affect electrostatic interaction between the kinase subunits. The region of the first domain that was changed due to the mutations (ribbon) is in yellow.

Exon 20 region is the same in the wild type (wt) and the modeled exon 19 LREA mutant (Figure 1A), but differs from the two modeled exon 20 mutants' possible structures (D770>GY, Figure 1B and D770_P772del_insKG, Figure 1C). Erlotinib has a similar position inside the wt or LREA mutant EGFR tyrosine kinase pocket. It is docked in the ATP-binding pocket without constraints (Figure 1A). When the mutation D770>GY is introduced, it can create significant changes in the conformation of the loop (yellow in Figure 1B). Its conformation is quite different in comparison with the wt loop (Figure 1A). This conformation can be stabilized by a hydrogen bond between the residues ASN772 and TYR828, π-cation interaction of ARG777 with TRP731, and hydrophobic interactions of VAL77 with LEU834 and VAL73, PRO81 with TYR136. Residue CYS275 located in the “back wall” of the ATP-binding pocket plays a role in positioning of incoming erlotinib. After a D770>GY mutation, residue CYS275 is located significantly farther from the drug's closest heavy atom. The absence of such a wall residue makes position of the drug in the pocket less defined (not having energy minimum in a proper position) and it can be significantly shifted. Such a shift can affect ATP competition for the binding site with the drug and activate the kinase despite erlotinib.

**Figure 2:**
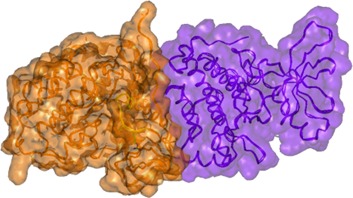
Surface presentation of the active dimer of the wt EGFR kinase subunits Note the close complementary interaction of the subunits.

**Figure 3:**
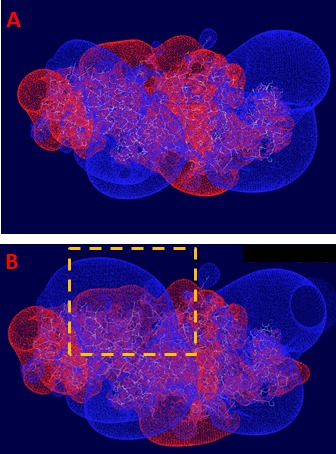
Electrostatic potentials profile of the kinase domains of EGFR dimer Blue—positive potential (+0.75e), red—negative (−0.75e). The mutated regions are outlined by the dashed square. The positive potential of the “left” kinase domains increases and extends closer to the negative potential of the “right” kinase domain. This fact causes increase of the attractive electrostatic energy of interaction: (A) WT EGFR; (B) D770_P772del_insKG mutant. The significant increase of positive electrostatic potential in the area of dimer interface is caused by the exon 20 insertion aberrations.

In the D770_P772del_insKG mutant, the new conformation of the loop (yellow in Figure 1C) is stabilized by the set of hydrophobic interactions. Specifically, interactions of: VAL769 with PHE855 and VAL765; VAL773 with ILE852; and CYS774 with MET766 and the hydrophobic “stem” of LYS851. The resulting loop has CYS775 also located quite far from the previous position with consequences similar to those described above for the D770>GY mutation. While the question of resistance of exon 20 mutants to the reversible EGFR inhibitors has been previously discussed [14, 15], the other issue as to why these mutations activate EGFR has not been well described. One of the explanations can be that these mutants can introduce some uncertainty in the position of ATP with changes in its phosphorylation potential. Another explanation can be related to changes in the electrostatic docking profile of the kinase's active dimer. The active configuration of this dimer is very tight and practically does not leave possible water enclaves (Figure 2). This fact increases the importance of possible electrostatic interactions between the domains that have a number of complementary positive and negative regions (Figure 3A).

**Figure 4:**
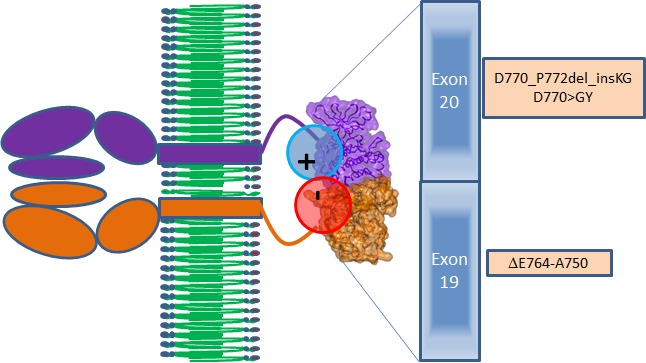
Scheme of interactions between the EGFR proteins in the active dimer (brown and violet) Positive–negative electrostatic interactions zone (red and blue circles) between the kinase domains of EGFR is caused by the mutations of exons 19 and 20 presented in the brown boxes.

We measured the electrostatic interactions between the two kinase domains of the EGFR dimer in active configuration and found that both mutations (D770_P772del_insKG and D770>GY) increase the attractive negative energy of inter-protein interaction (Table 1, Figure 4). The positive potential of the D770_P772del_insKG mutation region (outlined by dashed squares in Figure 3B) increases versus wild type (Figure 3A) with the subsequent increase in electrostatic interactions between the kinase domains of EGFR. This effect leads to “stabilization” of the dimeric active position. EGFR exists in equilibrium between the active and inactive dimers, and when it stays longer in the active dimer position, it would be more active.

**Table 1:** Electrostatic energy of interaction of the intracellular kinase domains in active conformation after mutations

Protein	ΔΔE (kJ/mol)
EGFR WT	0
EGFR T790M	0
EGFR D770_P772del_insKG	−93.93±10.5
EGFR D770>GY	−71.88±7.5
LREAdel exon 19 mutation	−60.54±6.3

### T790M mutant

**Figure 5:**
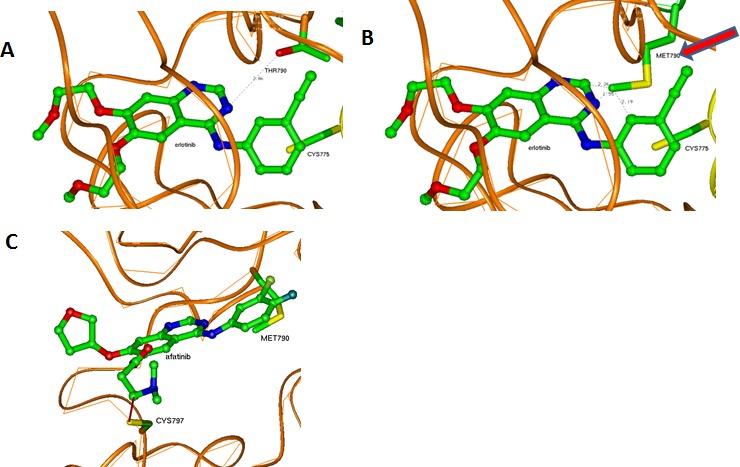
Interactions of erlotinib and afatinib in the ATP-binding pocket of EGFR kinases (A) Erlotinib in the wt or LREA mutant ATP-binding pocket. (B) Erlotinib in the T790M mutant ATP-binding pocket. Methionine 790 can prevent its proper positioning in the pocket causing instability and eventual departure because of an unstable position inside the pocket and competition with ATP. (C) Afatinib in the T790M mutant ATP-binding pocket. Covalent interaction with CYS797 makes it possible to stabilize the drug in the pocket.

The T790M mutation can result in enhanced affinity for ATP, and the subsequent activation is at least five fold compared to wild type [16]. In general, lung cancers caused by activating EGFR mutations are initially responsive to TKIs until second mutations, such as T790M, emerge and confer resistance by sterically blocking binding of TKIs [4]. Erlotinib docks to the ATP-binding pocket of EGFR without serious restraints in the case of the sensitive exon 19 LREA mutant (Figure 5A). The T790M mutation brings structural changes to the ATP-binding pocket (Figure 5B). Specifically, the bulky hydrophobic chain of MET790 that substitutes for THR790 (Figure 5B) will prevent the proper positioning of erlotinib and other reversible TKIs, introducing resistance to them. At the same time, the irreversible inhibitor afatinib, despite close resemblance to erlotinib's structure, inhibits the T790M mutant EGFR, at least in preclinical models (albeit with only minimal activity in the clinic) [17, 18]. Crystal structure of the kinase domain of EGFR with afatinib (Figure 5C) shows that it forces conformational changes in MET790 side chains [17]. A covalent bond between the drug and CYS797 causes this effect, making afatinib more stable in the binding position. When the side chain of MET790 is in the conformation adjusted to afatinib binding, that position would compete with ATP and allow afatinib to some extent to serve as a drug.

We checked the impact of T790M mutation on energy of interactions between the kinase domains of the active dimer of EGFR. We used the same method as for all abovementioned mutants. We found that there were no changes in electrostatic energy of the kinase domain interactions and consequently no changes of activity of EGFR (and no response to cetuximab) are predicted for that mutant (Table 1).

### LREA deletion mutant

**Figure 6:**
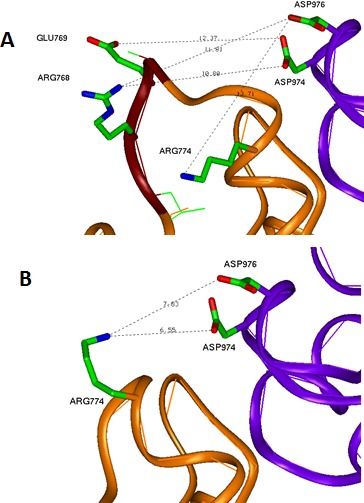
Conformational changes due to LREA mutation (A) WT EGFR interface between the kinase subunits in the region of exon 19. Negative GLU769 and positive ARG768 of the LREA loop region are located similar distances from the pair of negative aspartic acids ASP974 and ASP976, respectively, without significant impact to the electrostatic interaction between the subunits. (B) LREAdel mutant changes significantly the conformation of this loop and puts the positive ARG744 close to the above-mentioned pair of negative residues. This increases of the attractive energy of intersubunit interaction and leads to the more stable dimer state of the EGFR.

The del747–750 mutation in exon 19 activates EGFR. This mutation can significantly change the conformation of a protein in this region from the initial (Figure 6A) to the conformation with increased attractive electrostatic energy between the kinase domains of EGFR (Figure 6B, Table 1). This change can also prolong the “active dimer” configurational state. The effect of the LREA mutation on the active dimer stabilization can be less than that of the exon 20 aberrations D770_P772del_insKG and D770>GY because of positioning of the residues 747–750 close to the surface of the protein in the LREA mutation. The LREA mutation however may be sensitive to cetuximab too, albeit less so than the exon 20 insertion deletion mutants. Taking into account that the LREA mutant is known to be sensitive to the reversible TKI inhibitors, it may be that combination therapy (including an EGFR TKI inhibitor and EGFR antibody) can enhance response. Such a conclusion would, however, require a randomized study of an EGFR TKI versus an EGFR TKI combined with EGFR antibody, in order to be validated in patients. Based on this structural and electrostatic profile analysis, it would be predicted that when the somatic mutation T790M occurs, the combination therapy would no longer be effective because of resistance of T790M to reversible TKI.

### Patients

**Figure 7:**
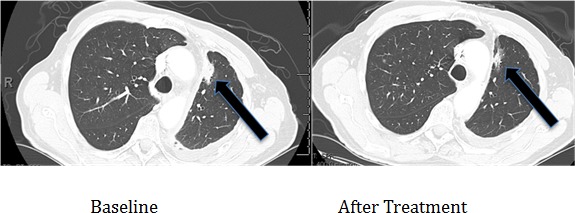
Computerized tomographic scan of the lungs of Patient #1 (Table 2) with EGFR aberration in exon 20 (D770>GY)) before and four months after treatment with a cetuximab-based regimen shows tumor regression

**Figure 8:**
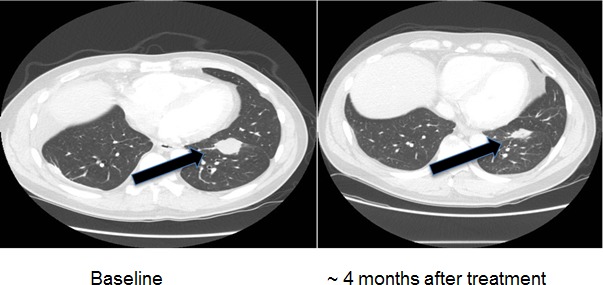
Computerized tomographic scan of the lungs of Patient #2 (Table 2) with EGFR exon 20 aberration D770_P772del_insKG before and after treatment with a cetuximab-based regimen shows tumor regression

Three patients with known EGFR-(reversible TKI) resistant mutations (two with insertions in exon 20 (Table 2, Cases 1 and 2) and one with an exon 20 T790M mutation (Case 3) were treated with an EGFR antibody (cetuximab)-based regimen. Both patients with insertions in exon 20 achieved durable partial remissions consistent with the predictions from our structural modeling (Table 2, Figures 7 and 8). The third patient, having the exon 19 (LREA-type) mutation, initially responded to the reversible TKI inhibitor erlotinib (partial remission for 17 months); at the time that the T790M was detected, the patient failed to respond to erlotinib combined with cetuximab, again consistent with the molecular structure predictions.

**Table 2:** Characteristics of patients with lung cancer, EGFR mutations and response to cetuximab-based therapy

Case No.	Age/Sex/Race	Diagnosis	Smoker Yes/No	EGFR Aberrations	Response to erlotinib alone	Response to cetuximab-based regimen	Comment
1	75/Male/Caucasian	Adenocarcinoma of the lung with pleural metastases	Yes (30 pack year)	exon 20 (D770>GY)	Unknown	Partial response, still progression-free at 42 months (Figure 7)	Received cetuximab with erlotinib
2	38/Male/Asian	Adenocarcinoma of the lung with bone metastases	No	D770_P772del_insKG in exon 20	Unknown	Partial response, still progression free at 6 months (Figure 8)	Received cetuximab with chemotherapy and bevacizumab
3	65/male/Asian	Adenocarcinoma of the lung with pleural, bones, and brain metastases	No	Two mutations: Deletion in exon 19 (15 base pair deletion (codons 746–750) (nested around LREA string at 747 to 750)) T790M in exon 20	Partial remission for 17 months, then progression (at time that T790M detected)	Rapid progression on cetuximab combined with erlotinib (given after progression on erlotinib, at time that T790M mutation was detected)	

## DISCUSSION

Our findings demonstrate that the exon 20 mutations EGFR D770_P772del_insKG and D770>GY (both resistant to EGFR reversible TKIs) [3] and exon 19 LREA del (EGFR reversible TKI-sensitive) [1], but not T790M (reversible TKI-resistant) [4, 5], can activate EFGR by increasing the attractive electrostatic energy between the monomer subunits of its kinase domains. The more attractive energies of interaction between the EGFR subunits would lead to more time that the receptor would stay in the active dimer conformation (Figure 1, Table 1). A question that could be raised is whether the values of changes of electrostatic energy are significant enough to cause changes in stability of the dimer (Table 1). To address this question, we reviewed the energies of inter-molecular interactions in known dimers of other proteins having dimensions that are close to those of the EGFR kinase domains. For the Ras–Raf dimer, the experimental value of inter-protein binding free energy is −40.2 kJ/mol [19] and the theoretical value is −62.8+26.4 kJ/mol [20]. (For the Ras–RalGDS, the experimental and theoretical values are correspondingly −35.2 kJ/mol [19] and −81.6 +24.7 kJ/mol [20].) For the “specially designed for electrostatic interaction” complex of barnase and barstar the calculated electrostatic energy is −61.9 kJ/mol [21]. Therefore, our values of electrostatic attractive energies from −60 to −93 kJ/mol (Table 1) are comparable with energies that are responsible for stability of other protein complexes.

The results of our structural and electrostatic profile analysis suggest that an antibody such as cetuximab, which functions by preventing receptor dimerization, might be effective in exon 20 insertion aberrations and, to a lesser extent, in LREA exon 19 mutants. Cetuximab binds to EGFR domain III and covers an epitope that partially overlaps with the ligand-binding site. The heavy chain of cetuximab also sterically prevents domain I of EGFR from adopting the conformation required for dimerization [22-24].

Of interest in this regard, we report three patients treated with EGFR antibody cetuximab-based regimens (Table 2). Two of these patients had mutations considered resistant to EGFR TKI inhibitors (D770>GY and D770_P772delsinKG (both in exon 20)), yet attained partial remissions on cetuximab-based therapy (Figures 7 and 8). One patient continues to do well on treatment for over 3.5 years (Table 2, Patient #1, and Figure 7). In contrast, a patient with the exon 20 T790M EGFR-TKI resistant mutation (in addition to the exon 19 sensitive LREAdel aberration) did not respond to the combination of cetuximab and erlotinib (Table 2, Patient #3). Typically, these patients present with the sensitive EGFR LREAdel aberrations and initially respond to reversible TKIs such as erlotinib (and, a salutary effect of erlotinib was indeed seen in our patient early in the disease course), but the resistant T790M mutation then emerges [4]. Similarly Janjigian and colleagues failed to show activity for the erlotinib/cetuximab combination in patients with NSCLC and acquired resistance to erlotinib due to T790M mutations [25]. Our clinical observations are supported by the *in silico* modeling data (Figures 1 and 5), which demonstrate that exon 20 insertion aberrations would be expected to increase the attractive electrostatic dimerization energies, and such a change could be predicted to predispose to response to an EGFR antibody that attenuates or interferes with dimerization. Further, our modeling suggests that the LREA mutation can react to the combination of cetuximab and erlotinib, but the combination of LREA and T790M mutations can compromise this effect because of resistance of T790M mutant to TKI. Although there are limitations to this study, e.g., the small number of patients and the fact that the responders received combination therapy, the expected rate of response to the other drugs in the combination is low. These data suggest that some patients with gefitinib/erlotinib-resistant EGFR mutations, especially those in the amino acid 770 region of exon 20, may benefit from therapy with EGFR antibodies.

Recently, several reports have postulated that the molecular heterogeneity and complexity of signaling in metastatic cancer can be distilled down to several pathways by analysis of convergence, interactions, and hubs [26-29]. While identifying convergence pathways is without a doubt important, it is also increasingly apparent that distinct mutations in the same gene or pathway can have vastly different effects, and real-time *in silico* modeling may therefore be an important tool for precision therapy. The current proof-of-principle report demonstrates cetuximab response in patients with EGFR aberrations in exon 20 (D770_P772del_insKG and D770>GY); these aberrations are resistant to EGFR TKIs. The response was predicted by *in silico* modeling, because the modeling showed that these molecular abnormalities activate EGFR by increasing the stability of the active dimer, and cetuximab is an EGFR antibody that interferes with dimerization.

In conclusion, as analysis of oncogenic aberrations in patients with cancer is adopted in practice, there is often still an assumption that distinct abnormalities in the same gene produce a similar or identical effect. Yet the functional impact of distinct aberrations in the same gene may differ greatly. For instance, if a mutation increases interaction of the members of the dimer leading to activation of a kinase, the optimal treatment may be an antibody that interferes with dimerization. If, on the other hand, a mutation increases the activity of the kinase, facilitating a switch to an active conformation, the ideal treatment might be a compound that preserves the inactive conformation, etc. *In silico* modeling, such as that in the current proof-of-principle study, as well as computer-based, decision-making systems, are therefore likely to be needed for best understanding anomalies in multiple genes beyond EGFR. It will be worthwhile to perform larger studies in order to confirm these observations and to determine if *in silico* modeling can be more widely exploited to precisely match patients harboring EGFR and other aberrations with drugs, so that response can be optimized.

## MATERIALS AND METHODS

### Rationale for molecular modeling use in these studies

EGFR can form two different dimers. The first symmetric dimer is inactive, while the second, asymmetric dimer is active. EGFR dimerization activates the EGFR function. There are no direct residue-to-residue electrostatic contacts between the kinase domains of the active dimer, and electrostatic interactions are not considered as a main driving force of its formation. Indeed, the electrostatic interactions would not be the main reason for EGFR dimerization. Nevertheless in the active dimer there exists very close complementary contact between the kinase domains. Such a situation makes the dielectric constant in the interface zone comparable to the constant inside the protein. When such a condition arises, more distant than direct salt-bridge electrostatic interactions would became important. Mutations that increase electrostatic interactions between the kinase domains would keep the dimer in the active position longer and would increase the general activity of EGFR. To check this hypothesis, we measured the energy of electrostatic interaction between the kinase domains in the active EGFR dimer (for wt and mutated proteins). Structural modeling of EGFR kinase mutants has been used before for prediction of EGFR drug resistance to erlotinib and gefitinib [14] and cetuximab [30] based on calculations of energies of drug-protein interactions. Nevertheless, to our knowledge, there are no reports describing modeling of EGFR mutants' impact on this protein's dimerization and consequent activation, and the clinical implications in individual patients.

The mutations studied (D770_P772del_insKG, D770>GY, and del747–750 (LREA)) include charged residues. These mutations are not just substitutions of one amino acid to another. D770_P772del_insKG makes the region shorter by one amino acid; D770>GY, longer by one amino acid; LREA, shorter by four amino acids. In all these cases, such changes would affect the tertiary structure of the protein. To elucidate a molecular mechanism explaining the function of these mutations, we conducted modeling of EGFR tertiary structure, and measured the effect of the mutations on interactions between two kinase domains of the EGFR dimer in the active state.

### Theoretical methods

We used the crystal structure of the active dimer of the kinase domains of EGFR—PDB ID 2GS6 [31]. For defining afatinib's position in T790M EGFR, we used the structures with PDB IDs 4G5P and 4G5J [17]. For modeling of all mutants, we used the Homology program from the InsightII program package (Accelrys, San Diego, CA). For measurement of electrostatic energy between the EGFR subunits, we used the non-linear Poisson–Boltzmann method as presented in the DELPHI program using AMBER forcefield [32, 33]. The dielectric constants 4 for protein and 78.6 for water were used. The potential profiles were calculated with Swiss-PdbViewer4.0.1 (www.expasy.org/spdbv/).

### Patients

Structural modeling of EGFR mutants was used, along with results of *Clinical Laboratory Improvement Amendments* (CLIA)-approved molecular tests for EGFR (next generation sequencing or polymerase chain reaction (PCR)-based sequencing). The index patient (Table 2, Case 2) was assessed to predict EGFR antibody responsiveness; this patient had an insertion exon 20 mutation known to be resistant to EGFR small molecule reversible kinase inhibitors [3]. (Modeling suggested increased responsiveness to an EGFR antibody for the insertion exon 20 mutations, but not for T790M exon 20 mutation.) The patient was treated with an EGFR antibody-containing regimen and outcomes, including tumor regression, ascertained using RECIST measurements [34]. The results of a twenty patient trial using a cetuximab-based regimen for lung cancer was then retrospectively analyzed for patients with resistant EGFR mutations [35]. The outcomes of the two patients harboring such mutations (Table 2, Cases 1 and 3) were evaluated. The study was performed consistent with the Internal Review Board guidelines for the University of California San Diego and University of Texas MD Anderson Cancer Center (depending on where they were treated).
